# Insulin Resistance Increases MRI-Estimated Pancreatic Fat in Nonalcoholic Fatty Liver Disease and Normal Controls

**DOI:** 10.1155/2013/498296

**Published:** 2013-11-17

**Authors:** Niraj S. Patel, Michael R. Peterson, Grace Y. Lin, Ariel Feldstein, Bernd Schnabl, Ricki Bettencourt, Ekihiro Seki, Claude B. Sirlin, Rohit Loomba

**Affiliations:** ^1^Division of Internal Medicine, Department of Medicine, University of California at San Diego, UC San Diego Health System, 9500 Gilman Drive, MC 0063, La Jolla, CA 92093, USA; ^2^Department of Pathology, University of California at San Diego, UC San Diego Health System, 9500 Gilman Drive, MC 0063, La Jolla, CA 92093, USA; ^3^Division of Gastroenterology, Department of Pediatrics, University of California at San Diego, UC San Diego Health System, 9500 Gilman Drive, MC 0063, La Jolla, CA 92093, USA; ^4^Division of Gastroenterology, Department of Medicine, University of California at San Diego, UC San Diego Health System, 9500 Gilman Drive, MC 0063, La Jolla, CA 92093, USA; ^5^Division of Epidemiology, Department of Family and Preventive Medicine, University of California at San Diego, UC San Diego Health System, 9500 Gilman Drive, MC 0063, La Jolla, CA 92093, USA; ^6^Liver Imaging Group, Department of Radiology, University of California at San Diego, UC San Diego Health System, 9500 Gilman Drive, MC 0063, La Jolla, CA 92093, USA

## Abstract

*Background*. Ectopic fat deposition in the pancreas and its relationship with hepatic steatosis and insulin resistance have not been compared between patients with nonalcoholic fatty liver disease (NAFLD) and healthy controls. *Aim*. Using a novel magnetic resonance imaging (MRI) based biomarker, the proton-density-fat-fraction (MRI-PDFF), we compared pancreatic fat content in patients with biopsy-proven NAFLD to healthy controls and determined whether it is associated with insulin resistance and liver fat content. *Methods*. This nested case-control study was derived from two prospective studies including 43 patients with biopsy-proven NAFLD and 49 healthy controls who underwent biochemical testing and MRI. *Results*. Compared to healthy controls, patients with NAFLD had significantly higher pancreatic MRI-PDFF (3.6% versus 8.5%, *P* value <0.001), and these results remained consistent in multivariable-adjusted models including age, sex, body mass index, and diabetes (*P* value =0.03). We found a strong correlation between hepatic and pancreatic MRI-PDFF (Spearman correlation, *P* = 0.57, *P* value <0.001). Participants with increased insulin resistance determined by homeostatic-model-of-insulin-resistance (HOMA-IR) greater than 2.5 had higher pancreatic (7.3% versus 4.5%, *P* value =0.015) and liver (13.5% versus 4.0%, *P* value <0.001) MRI-PDFF. *Conclusion*. Patients with NAFLD have greater pancreatic fat than normal controls. Insulin resistance is associated with liver and pancreatic fat accumulation.

## 1. Introduction

It is well established that obesity, insulin resistance, and other components of metabolic syndrome play a role in the development and progression of nonalcoholic fatty liver disease (NAFLD) [1–3]. Due to increasing rates of obesity, the prevalence of NAFLD is increasing and now affects approximately 30% of the adult population in the western world [4, 5]. Most patients with NAFLD have a relatively benign course who are classified as having NAFL that rarely progresses to cirrhosis and is not associated with increased risk of liver-related morbidity and mortality. However, 10–20% of patients with NAFLD have the progressive form of NAFLD termed as nonalcoholic steatohepatitis (NASH) [6, 7], which can lead to cirrhosis and end-stage liver disease and is associated with increased liver-related morbidity and mortality [8].

 Similar to fat accumulation in the liver, obesity and metabolic syndrome result in ectopic fat deposition in other organ systems including skeletal muscles, the heart, and the pancreas. In the setting of metabolic syndrome, fat accumulation in the pancreas may lead to a similar process of inflammation, or “nonalcoholic steatopancreatitis” [9]. Recent studies have linked fatty pancreas to hepatic steatosis, obesity, insulin resistance and metabolic syndrome [10–15]. 

Although questions remain regarding the clinical implications of fat accumulation in the pancreas, fatty pancreas may become an increasingly relevant condition with the rising prevalence of NAFLD. Few studies have evaluated the relationship between hepatic steatosis and fat accumulation in the pancreas. A recent study by our group revealed that amongst patients with biopsy-proven NAFLD, histology-determined steatohepatitis and liver steatosis were associated with higher levels of magnetic resonance imaging (MRI) estimated fat content in the pancreas [16]. This study utilized an advanced chemical shift based gradient-echo MRI technique that measures the proton-density-fat-fraction (PDFF), a quantitative marker of fat content in tissue [17]. One limitation of this study was the lack of a control group. In addition, the effect of fatty pancreas on insulin resistance remains unclear. A comparison of pancreatic MRI-PDFF between patients with NAFLD and healthy controls has not been previously studied.

In this study we aim to determine whether pancreatic MRI-PDFF is greater in patients with NAFLD than healthy controls. We also explore the relationship between components of metabolic syndrome including insulin resistance and pancreatic fat content. These insights may help us understand whether pancreatic steatosis is a marker of metabolic syndrome and liver steatosis and whether it is a risk factor for progression of NAFLD.

## 2. Methods

### 2.1. Study Design and Patient Population

This is a nested case-control study derived from two prospective studies of healthy individuals and patients with biopsy-proven NAFLD at the UCSD NAFLD Research Unit (http://fattyliver.ucsd.edu/). 43 consecutive adult participants with biopsy-proven NAFLD and 49 consecutive health control participants underwent clinical research evaluation, physical examination, biochemical testing, and detailed MRI phenotyping. All patients with NAFLD were diagnosed by liver biopsy as well as exclusion of other causes of liver disease (as detailed in the following section). All patients provided written informed consent to participate in the study and the study was approved by the University of California San Diego Institutional Review Board. All patients underwent a standard history and physical exam, biochemical testing, and MRI examination at UCSD. They also all underwent an alcohol history assessment by completing the AUDIT and Skinner Lifetime Drinking questionnaires.

### 2.2. Inclusion and Exclusion Criteria

#### 2.2.1. NAFLD Cohort

*Definition of NAFLD.* (1) Fat accumulation in the liver (steatosis) involves at least 5% of hepatocytes on routine stains. (2) No evidence of other acute or chronic liver disease was found. (3) There was absence of regular or excessive use of alcohol within 2 years prior to entry.

Inclusion criteria in the NAFLD cohort included (1) age greater than 18 years; (2) evidence of NAFLD (as described above) on liver biopsy as assessed by the NASH-CRN histologic scoring system [18]; (3) ability and willingness to give written, informed consent to be screened for and, if eligible, to be enrolled into the NAFLD Cohort Study. Exclusion criteria included (1) clinical or histological evidence of alcoholic liver disease: regular and excessive use of alcohol within the 2 years prior to interview defined as alcohol intake greater than 14 drinks per week in a man or greater than 7 drinks per week in a woman; approximately 10 g of alcohol equals one “drink” unit; one unit equals 1 ounce of distilled spirits, one 12 oz beer, or one 4 oz glass of wine; the AUDIT and Skinner Lifetime Drinking questionnaires were utilized to systematically assess alcohol use in the population; (2) total parenteral nutrition for more than 1 month within a 6 month period before baseline liver biopsy; (3) short bowel syndrome; (4) history of gastric or jejunoileal bypass preceding the diagnosis of NAFLD; (5) bariatric surgery performed following enrollment is not exclusionary; Liver biopsies obtained during bariatric surgery cannot be used for enrollment because of the associated surgical or anesthetic acute changes and the weight loss efforts that precede bariatric surgery; (6) history of biliopancreatic diversion; (7) evidence of advanced liver disease defined as a Child-Pugh-Turcotte score equal to or greater than 7; (8) evidence of chronic hepatitis B as marked by the presence of HBsAg in serum (participants with isolated antibody to hepatitis B core antigen, anti-HBc total, are not excluded); (9) evidence of chronic hepatitis C as marked by the presence of anti-HCV or HCV RNA in serum; (10) low alpha-1-antitrypsin level and ZZ phenotype (both determined at the discretion of the investigator); (11) wilson's disease. (12) known glycogen storage disease or dysbetalipoproteinemia; (13) known phenotypic hemochromatosis (HII greater than 1.9 or removal of more than 4 g of iron by phlebotomy); (14) prominent bile duct injury (florid duct lesions or periductal sclerosis) or bile duct paucity; (15) chronic cholestasis; (16) vascular lesions (vasculitis, cardiac sclerosis, acute or chronic Budd-Chiari, hepatoportal sclerosis, or peliosis); (17) concomitant severe underlying systemic illness that in the opinion of the investigator would interfere with completion of followup; (18) inability to undergo MRI.

#### 2.2.2. Healthy (Non-NAFLD) Cohort

Inclusion criteria in the healthy (non-NAFLD) control group included (1) age greater than 18 years and (2) liver MRI-PDFF <5%. A threshold of 5% is consistent with magnetic resonance spectroscopy (MRS) determined NAFLD [19]. We have previously shown a robust correlation of 0.99% between liver MRI-PDFF and liver MRS-PDFF and validated the use of MRI-PDFF as biomarker for liver fat quantification [16, 20–23]. They also have (3) ability and willingness to give written, informed consent to be screened for and, if eligible, to be enrolled into the NAFLD Cohort Study. Exclusion criteria included (1) serum alanine aminotransferase (ALT) or aspartate aminotransferase (AST) levels above the upper limit of normal (19 U/L or more for women and 30 U/L or more for men), (2) significant systemic illness, (3) no serologic or biochemical evidence of chronic liver disease or past history of treatment for acute or chronic liver disease, (4) negative viral serologies (HBsAg and anti-HCV) and iron profile, (5) clinical evidence of excessive alcohol use as defined above (see NAFLD cohort exclusion criteria), and (6) inability to undergo MRI.

### 2.3. Clinical Evaluation

After meeting inclusion and exclusion criteria, patients underwent a routine history and physical exam in a research clinic. Body weight, height, and vital sign measurements were obtained and standard blood testing was performed, including measurement of ALT, AST, alkaline phosphatase, gamma-glutamyl transpeptidase (GGT), total bilirubin, direct bilirubin, albumin, fasting glucose and insulin, hemoglobin A1c (HbA1c), lipid panel, free fatty acids (FFA), and C-reactive protein (CRP). Homeostatic-model-of-insulin-resistance (HOMA-IR) was calculated as the product of fasting insulin and glucose divided by a correction factor of 405.

### 2.4. MRI Protocol

In order to quantify pancreas and liver fat content, we used a previously described advanced chemical shift based gradient-echo MRI technique that estimates PDFF, which is a standardized and objective measure of fat content [16, 17, 20, 21, 23, 24]. It acquires multiple echo sequences at different times with fat and water signals nominally in phase or out of phase with each other and applies an algorithm to generate a PDFF parametric map depicting fat quantity and distribution throughout the pancreas and liver. This method is independent of scanner platform, manufacturer, and other factors that may affect fat content measurements made by conventional MRI techniques. It has been shown to reliably measure pancreatic fat content when compared to other MRI techniques [25]. In addition, it accurately measures liver fat fraction when compared to MRS [26] and is more sensitive than histology-determined steatosis grade [21]. 

In order to estimate PDFF across the entire liver, 3 regions of interest (ROIs) of 300 mm^2^ to 400 mm^2^ in area were placed in each of the nine liver segments on the PDFF parametric maps. Similarly, pancreatic PDFF was measured by placing 1 to 2 ROIs of 100 mm^2^ each in the head, body, and tail of the pancreas in each slice of the PDFF parametric maps. These protocols have been described in prior studies [16, 20, 24]. The mean of all ROIs in the liver and pancreas was calculated to determine the average PDFF in each organ. 

A single resident physician who was trained in this method of MRI analysis performed the measurements. The physician was blinded to clinical and histological data and was under the supervision of the radiology investigator (CS). These findings were cross-validated by an independent radiology investigator who was blinded to the prior pancreatic and liver fat fraction maps.

### 2.5. Statistical Analysis

The two-tailed *t*-test was used for comparison of continuous variables between the NAFLD and control groups, while the chi-square test was used for comparisons of categorical variables. A multivariable-adjusted linear regression model was used to compare liver MRI-PDFF and pancreatic MRI-PDFF between groups after adjusting for differences in age, sex, body-mass index (BMI), and diabetes between the groups. A Spearman correlation was performed to compare MRI-PDFF of the liver and pancreas amongst all patients. Sample size estimation: we hypothesized that MRI-PDFF of pancreas would positively correlate with MRI-PDFF of liver, and therefore, pancreatic fat content would be higher in participants with NAFLD versus normal controls. We would need a sample size of at least 40 to have an alpha of 0.05 with a power of 80% (or higher) requiring an effect size of 0.38 or higher. All the statistical analysis was performed using Excel and SPSS software packages (Released 2009. PASW Statistics for Windows, Version 18.0. Chicago: SPSS Inc). In all analyses, *P*-value <0.05 was considered statistically significant.

## 3. Results

### 3.1. Demographic and Biochemical Data of Patients: NAFLD versus Healthy Controls

Forty-three patients with biopsy confirmed NAFLD and 49 healthy controls were enrolled in this study between 1/2010 and 3/2013. Demographic and biochemical data for these patients are shown in Table 1. Patients in the NAFLD group were slightly older than healthy controls (mean ± standard deviation; 48.4 years ± 11.9 versus 43.2 years ± 20.0); however this difference was not significant. A significantly higher proportion of NAFLD patients were male compared to healthy controls (55.8% versus 22.5%, *P*-value <0.001). As expected, NAFLD patients had a higher BMI (in kg/m^2^) than controls (31.5 ± 4.6 versus 25.5 ± 7.2, *P*-value <0.001). Other metabolic parameters are also provided in Table 1.

### 3.2. MRI Estimated Pancreatic Fat and Liver Fat Content: NAFLD versus Healthy Controls

**Figure 1:**
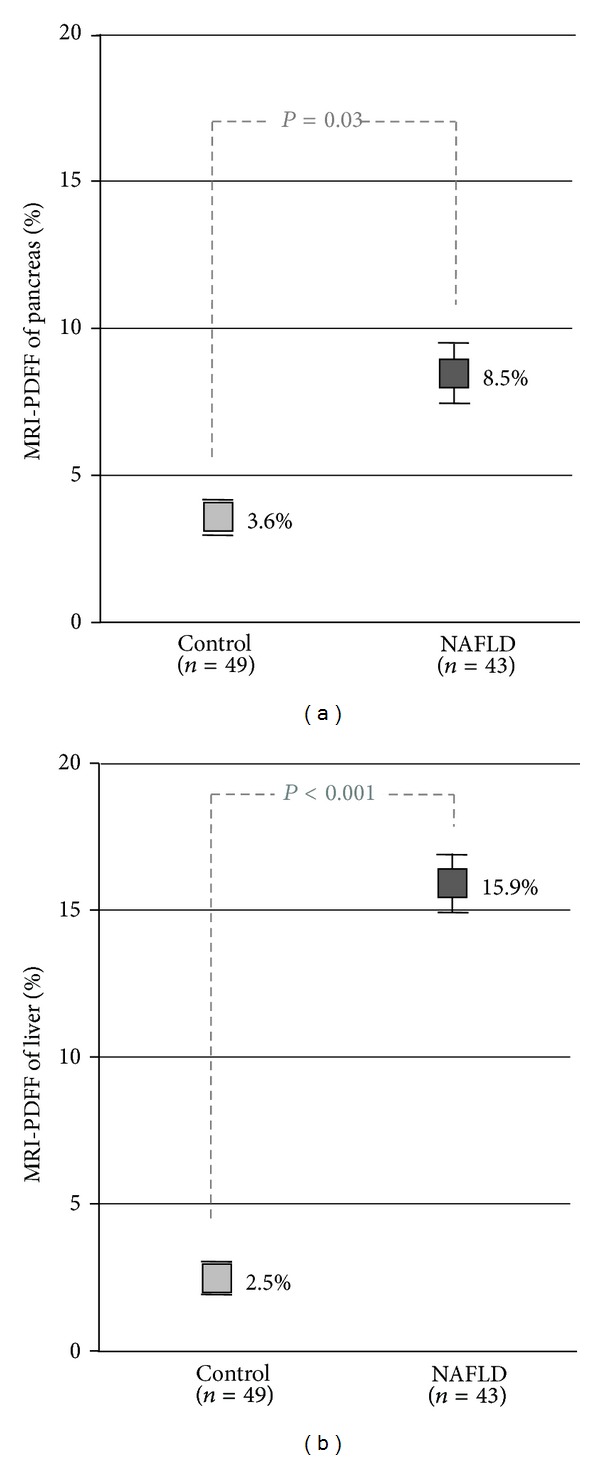
MRI-estimated pancreatic and liver fat content in healthy controls versus NAFLD patients. Mean magnetic resonance imaging (MRI) proton-density-fat-fraction (PDFF) is shown for the pancreas and liver. Standard error bars are shown. *P*-value, determined using multivariable linear regression to correct for differences in age, sex, body mass index (BMI), and diabetes, is shown.

**Figure 2:**
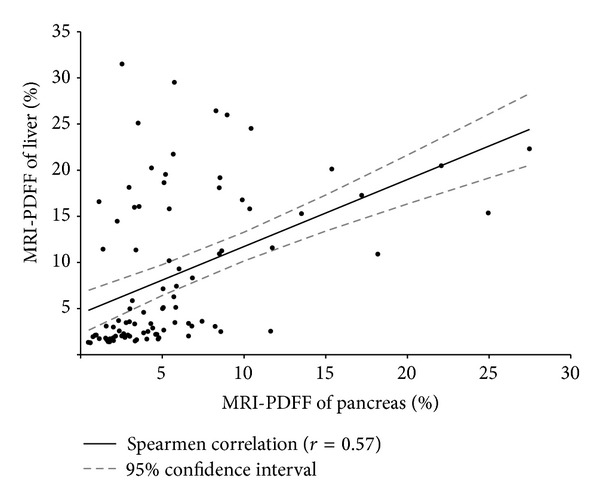
Correlation of MRI-estimated pancreatic fat and liver fat content. Magnetic resonance imaging (MRI) proton-density-fat-fraction (PDFF) of liver (*y*-axis) and pancreas (*x*-axis) are shown for all patients in control and NAFLD groups. Spearman correlation coefficient determined with regression line and 95% confidence limits (*r* ^2^ = 0.32) are shown.

MRI-estimated pancreatic fat content was significantly greater in patients with NAFLD than healthy controls (8.5% ± 6.6 versus 3.6% ± 2.3, *P*-value <0.001) as shown in Figure 1. Multivariate statistical analysis revealed that this difference remained significant after adjusting for differences in age, sex, BMI and diabetes status between these groups (*P*-value =0.03). As expected, MRI-estimated liver fat content was significantly greater in patients with NAFLD than healthy controls (15.9% ± 6.7 versus 2.5% ± 0.9, *P*-value <0.001). MRI-PDFF of the liver had a significant correlation with MRI-PDFF of the pancreas (Spearman correlation coefficient of 0.57, *P*-value <0.001) as shown in Figure 2.

### 3.3. Insulin Resistance: NAFLD versus Healthy Controls

**Figure 3:**
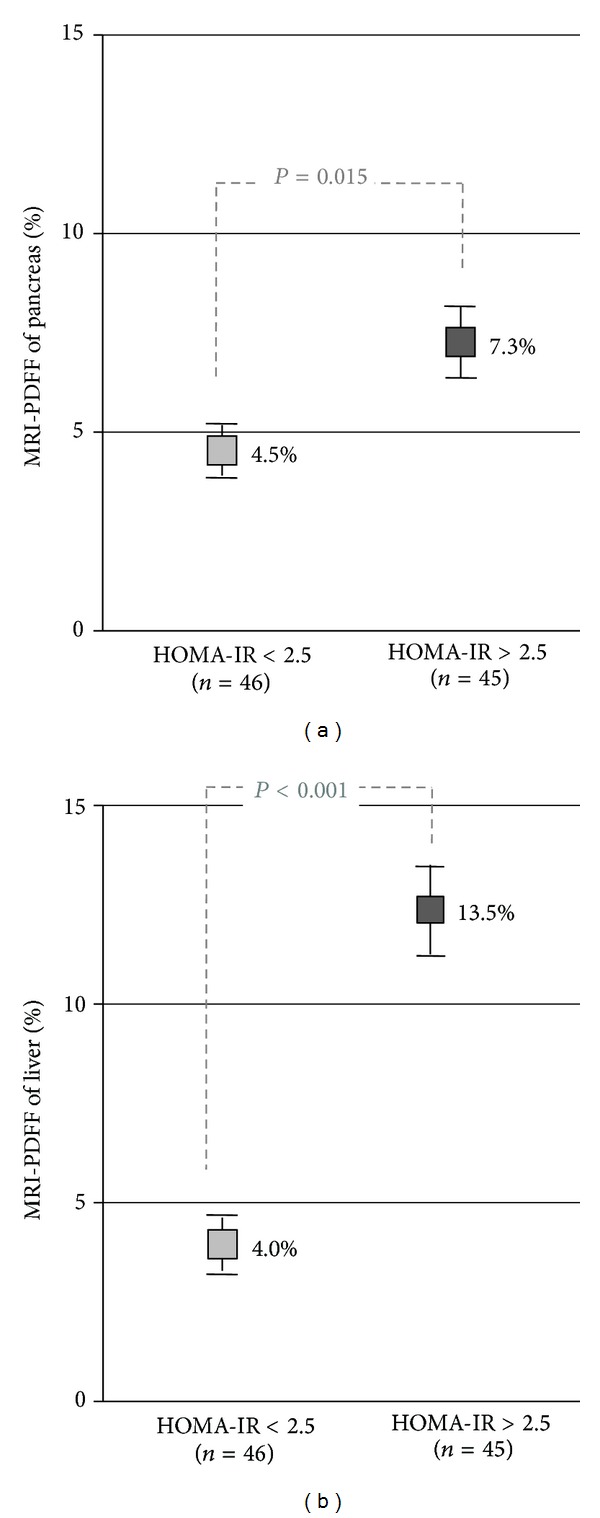
MRI-estimated pancreatic and liver fat content versus insulin resistance. Mean magnetic resonance imaging (MRI) proton-density-fat-fraction (PDFF) of pancreas and liver is shown for all patients in control and NAFLD groups. Participants with homeostatic-model-of-insulin-resistance (HOMA-IR) score less than the median value of 2.5 compared to participants with HOMA-IR greater than 2.5. *P*-value determined using a two-tailed *t*-test.

Insulin resistance determined by HOMA-IR varied from 0.4 to 63.0 (median = 2.5) amongst all patients. Participants with increased insulin resistance determined by HOMA-IR greater than 2.5 (above the median HOMA-IR) had higher MRI-PDFF estimated pancreatic fat (7.3% versus 4.5%, *P*-value =0.015) and liver fat (13.5% versus 4.0%, *P*-value <0.001) than those below the median HOMA-IR (please see Figure 3). 

## 4. Discussion

In this nested prospective case control study using an advanced, validated MRI-method that allows noninvasive fat quantification of the pancreas and liver, we demonstrate that patients with biopsy-proven NAFLD have higher pancreatic fat content than healthy controls. This difference was confirmed after multivariate analysis correcting for differences in risk factors of metabolic syndrome between these two groups. Furthermore, there is a good correlation between MRI-estimated liver fat and pancreatic fat. In addition, similar to liver fat, insulin resistance was associated with increased pancreatic fat in this cohort, suggesting shared genetic and environmental effects [27, 28]. In summary, these findings confirm a strong relationship between liver and pancreatic fat content and suggest that insulin resistance is a risk factor for and/or result of non-alcoholic fatty pancreas.

Risk factors for pancreatic fat deposition have been studied in the past using multiple modalities. One of the limitations of assessment of fatty pancreas is the inability to obtain an in vivo biopsy. Early studies used postmortem histologic analysis to show a relationship between pancreatic fat and increased age, obesity, and adult-onset diabetes [29, 30]. A more recent postmortem study by van Geenen et al. revealed that histology-determined pancreatic fat and liver fat were related and that intralobular pancreatic fat in particular is associated with NASH [31]. Ultrasonography is a relatively insensitive measure of fat content; however, prior studies identified a relationship between fatty pancreas estimated by ultrasonography and increased age, dyslipidemia, obesity, and insulin resistance [13, 32]. In a study of healthy patients, Wu and Wang determined that fatty pancreas diagnosed with ultrasonography was associated with multiple components of metabolic syndrome and suggest that this disease entity is a meaningful manifestation of metabolic syndrome [10]. In addition, Choi et al. and Al-Haddad et al. found a strong relationship between ultrasonography-estimated pancreatic fat and hepatic steatosis in cohorts of patients undergoing endoscopic ultrasound [11, 15]. 

More recently, MRI techniques have been utilized for the assessment of pancreatic fat deposition. Li et al. used fat emulsions to validate a chemical shift gradient-echo MRI technique to measure pancreatic fat fraction and confirmed that fat content increased with aging [33]. Targher et al. noted that pancreatic fat content was associated with liver fat and insulin resistance in a cohort of obese patients with NAFLD [34]. In addition, a recent study by our group showed that pancreatic fat content estimated by MRI is associated with histology-determined steatosis grade in patients with NAFLD [16]. 

Our analysis confirms findings from prior studies that suggest an association between pancreatic and liver fat accumulation. Unlike prior studies, a comparison between a healthy control group and patients with biopsy-proven NAFLD was used to determine a relationship between NAFLD and fatty pancreas that is independent of obesity, diabetes, and age. In addition, the strong correlation between pancreatic MRI-PDFF and liver MRI-PDFF suggests that regardless of the presence of NAFLD, pancreatic fat may be a marker for ectopic fat deposition in other organs.

Our study clearly demonstrates an association between increased insulin resistance and fatty pancreas. Prior studies have established that insulin resistance is a risk factor for the development of NAFLD and may be associated with advanced disease [35–37]. In particular, diabetes and worsening of metabolic factors have been linked to the development of NASH and advanced fibrosis in patients with NAFLD [38–40]. Fatty pancreas may potentiate metabolic syndrome by resulting in beta cell dysfunction and hyperglycemia [41]. Tushuizen et al. noted that this relationship may lead to the development of diabetes in susceptible individuals [42]. In addition, impaired pancreatic beta cell function in particular has been linked to NASH amongst patients with hepatic steatosis [43]. Pancreatic fat may be a marker of metabolic syndrome and is associated with diabetes, independent of other risk factors [10, 32, 44]. 

The relationship between pancreatic fat, liver fat, and insulin resistance noted in our study and prior studies leads to an important question regarding the effect of fatty pancreas on the development and progression of NAFLD. Based on our findings, we propose that, similar to NAFLD, fatty pancreas may be an end-result of insulin resistance and metabolic syndrome. Conversely, it also may be a risk factor for the development of metabolic syndrome, which can lead to ectopic fat deposition in the liver and increase the risk of the developing NASH and advanced fibrosis. This proposed relationship suggests that pancreatic fat potentiates insulin resistance and therefore may lead to the development and progression of NAFLD.

### 4.1. Strengths and Limitations

The major strengths of this study include the use of a well-characterized patient population with biopsy-proven NAFLD and the inclusion of a uniquely phenotyped control group using MRI-PDFF. Ultrasound is insensitive in differentiating normal from NAFLD and a liver biopsy is unethical in normal individuals. Therefore, MRI is needed to accurately classify a participant as having a normal liver (with less than 5% liver fat content) for comparison with participants who have NAFLD. Most prior noninvasive studies of pancreatic fat reviewed cohorts of obese or healthy individuals without known NAFLD. In addition, this study utilized an MRI technique that has been well validated to measure fat content in the liver and has been used previously to measure fat content in the pancreas. Although this was a nested case control study in which individual patients were not matched with healthy controls, the use of multivariate statistics allowed analysis of liver and pancreatic fat content between the two groups independent of differences noted between these groups. Despite this, we do acknowledge limitations of this study. As this is not a longitudinal study, we are unable to assess whether pancreatic fat affects progression of NAFLD. In addition, although the MRI technique used in this study has been well validated in the liver, it has not been validated in analysis of pancreatic tissue. Finally, only NAFLD patients had histologic analysis, precluding the ability to compare histology in NAFLD patients to healthy controls in this study. Due to the risks of liver biopsy, it was not ethically possible to pursue a liver biopsy on healthy control participants.

### 4.2. Implications for Future Research

Additional studies are needed in this area to further characterize the relationship between pancreatic and liver fat. Longitudinal studies should focus on the effect of pancreatic fat on progression of NAFLD and development of NASH and/or worsening of insulin resistance or fibrogenesis in the pancreas itself. In addition, the development of fatty pancreas independent of NAFLD should be evaluated as a biomarker of metabolic syndrome and a risk factor for the development and progression of NAFLD.

## 5. Conclusions

 Patients with biopsy-proven NAFLD have higher pancreatic fat content than healthy controls. In addition, there is a significant correlation between MRI-estimated pancreatic and liver fat content amongst patients with NAFLD and healthy controls. Increased insulin resistance determined by HOMA-IR is associated with increased liver and pancreatic fat content. Future studies are needed to determine the effect of pancreatic fat on development and progression of NAFLD.

## Figures and Tables

**Table 1:** Demographic and biochemical characteristics of patients with NAFLD and healthy controls.

	NAFLD patients (*n* = 43)	Healthy controls (*n* = 49)	*P* value
Age (years)	48.4 (11.9)	43.2 (20.0)	0.14
Sex (% male)	55.8%	22.5%	<0.001
BMI (kg/m^2^)	31.5 (4.6)	25.5 (7.2)	<0.001
Diabetes (%)	34.9%	32.7%	0.74
AST (U/L)	53.7 (42.4)	21.0 (6.5)	<0.001
ALT (U/L)	82.3 (61.8)	18.8 (11.0)	<0.001
Glucose (mg/dL)	110.0 (29.9)	88.7 (9.9)	<0.001
Insulin (*µ*IU/mL)	29.3 (36.2)	7.5 (4.4)	<0.001
HOMA-IR	8.6 (11.7)	1.7 (1.0)	<0.001
Hgb A1c (%)	6.29 (0.90)	5.60 (0.26)	<0.001
Triglycerides (mg/dL)	180.3 (125.8)	78.6 (45.6)	<0.001
Total cholesterol (mg/dL)	200.7 (43.8)	183.2 (32.3)	0.03
LDL (mg/dL)	120.3 (36.7)	100.8 (26.7)	0.004
HDL (mg/dL)	47.6 (16.2)	66.8 (19.9)	<0.001
Alk Phos (U/L)	78.3 (23.2)	66.9 (22.9)	0.02
GGT (U/L)	73.4 (65.5)	19.2 (11.2)	<0.001
Total bilirubin (mg/dL)	0.59 (0.39)	0.46 (0.22)	0.05
MRI-PDFF pancreas (%)	8.5% (6.6)	3.6% (2.3)	<0.001
MRI-PDFF liver (%)	15.9% (6.7)	2.5% (0.9)	<0.001

Data expressed as mean with standard deviation in parentheses. Abbreviations for tables: NAFLD: nonalcoholic fatty liver disease; BMI: body mass index; AST: aspartate aminotransferase; ALT: alanine aminotransferase; HOMA-IR: homeostatic-model-of-insulin-resistance; Hgb A1c: hemoglobin A1c; LDL: low-density lipoprotein; HDL: high-density lipoprotein; Alk Phos: alkaline phosphatase; GGT: gamma-glutamyl transpeptidase; MRI: magnetic resonance imaging; PDFF: proton-density-fat-fraction. Insulin levels were measured while fasting. *t*-test assuming equal variance between NAFLD and control group.

## Abbreviation

MRI: Magnetic resonance imaging.
